# Therapeutic effects of adipose-tissue-derived mesenchymal stromal cells and their extracellular vesicles in experimental silicosis

**DOI:** 10.1186/s12931-018-0802-3

**Published:** 2018-05-29

**Authors:** Elga Bandeira, Helena Oliveira, Johnatas D. Silva, Rubem F. S. Menna-Barreto, Christina M. Takyia, Jung S. Suk, Kenneth W. Witwer, Michael E. Paulaitis, Justin Hanes, Patricia R. M. Rocco, Marcelo M. Morales

**Affiliations:** 1 Carlos Chagas Filho Biophysics Institute, Federal University of Rio de Janeiro, Rio de Janeiro, Brazil; 2 Oswaldo Cruz Institute, Oswaldo Cruz Foundation, Rio de Janeiro, Brazil; 3 Center for Nanomedicine, The Johns Hopkins University School of Medicine, Baltimore, MD USA; 4 Departments of Molecular and Comparative Pathobiology and Neurology, The Johns Hopkins University School of Medicine, Baltimore, MD USA; 5 Laboratory of Cellular and Molecular Physiology, Carlos Chagas Filho Institute of Biophysics, Federal University of Rio de Janeiro, Centro de Ciencias da Saude, Avenida Carlos Chagas Filho, s/n, Bloco G1-55, Ilha do Fundao, Rio de Janeiro, RJ 21941-902 Brazil

**Keywords:** Silicosis, Mesenchymal stromal cells, Inflammation, Fibrosis, Extracellular vesicles

## Abstract

**Background:**

Silicosis is an occupational disease that affects workers who inhale silica particles, leading to extensive lung fibrosis and ultimately causing respiratory failure. Mesenchymal stromal cells (MSCs) have been shown to exert therapeutic effects in lung diseases and represent an alternative treatment for silicosis. Recently, it has been suggested that similar effects can be achieved by the therapeutic use of extracellular vesicles (EVs) obtained from MSCs. The aim of this study was to investigate the effects of adipose-tissue-derived MSCs (AD-MSCs) or their EVs in a model of silicosis.

**Methods:**

Silicosis was induced by intratracheal instillation of silica in C57BL/6 mice. After the onset of disease, animals received saline, AD-MSCs, or EVs, intratracheally.

**Results:**

At day 30, AD-MSCs and EVs led to a reduction in collagen fiber content, size of granuloma, and in the number of macrophages inside granuloma and in the alveolar septa. In addition, the expression levels of interleukin 1β and transforming growth factor beta in the lungs were decreased. Higher dose of EVs also reduced lung static elastance when compared with the untreated silicosis group.

**Conclusions:**

Both AD-MSCs and EVs, locally delivered, ameliorated fibrosis and inflammation, but dose-enhanced EVs yielded better therapeutic outcomes in this model of silicosis.

**Electronic supplementary material:**

The online version of this article (10.1186/s12931-018-0802-3) contains supplementary material, which is available to authorized users.

## Background

Silicosis is an occupational lung disease caused by the inhalation of small crystalline silica particles. The disease is characterized by granuloma formation and massive lung fibrosis, leading to respiratory failure. Silicosis remains a major concern in developing countries, despite remarkable progress in silicosis prevention in industrialized countries [1]. Currently, there are no clinically available treatments to revert or halt silicosis progression [2].

Cell therapy, including therapies based on the transplantation of mesenchymal stromal cells (MSCs), can both promote immunomodulation and affect remodeling, and therefore have been studied extensively as a therapeutic approach in a wide variety of respiratory diseases, including silicosis [3–5]. Progress toward silicosis cell therapy includes a recent phase I clinical trial using bone marrow cells [6]. In murine model studies, cell therapy reduced lung fibrosis and granuloma size, as well as pro-inflammatory and pro-fibrogenic mediators related to silicosis pathophysiology [7–10]. Cell therapy has limitations, however, including invasive cell collection procedures and the multiple dosing needed to sustain therapeutic effects [8, 9]. Furthermore, there are safety concerns about the potential of transplanted cells to proliferate and differentiate. However, therapeutic potency has also been achieved through the use of cell culture supernatant from MSCs, suggesting that cell therapy may act, at least in part, through endocrine and paracrine effectors [11, 12].

Recently, it has been suggested that extracellular vesicles (EVs) obtained from the conditioned media of MSCs may have similar effects to the cells of origin [13–16]. “Extracellular vesicles” is a term that comprises different membrane vesicles ubiquitously secreted by cells. Among them are exosomes (vesicles that can be found in ~ 100,000×*g* ultracentrifugation pellets, which are released in bulk when multivesicular bodies fuse with the plasma membrane), and microvesicles (larger vesicles up to 1 μm in diameter that are formed by direct evagination of the plasma membrane). EVs can carry small, messenger, and other RNAs, proteins, lipids, and organelles that can promote changes in gene expression and the behavior of target cells [17]. EV-based therapy could bypass some cell-therapy-associated safety concerns and reduce the need for repeated invasive procedures. Furthermore, EVs could be artificially improved or loaded to achieve enhanced effects [18–23]. However, current challenges with developing EV-based therapies are the lack of standardized approaches to EV isolation [24] and the need for clarification of the pharmacological properties and mechanisms of action of EVs.

In this study, we have characterized EVs obtained from adipose-tissue-derived mesenchymal stromal cells (AD-MSCs) and investigated the therapeutic impact of AD-MSCs or EVs produced by these cells in inflammation and remodeling as an alternative to cell-based therapy for silicosis.

## Methods

### Animal preparation and experimental protocol

This study was approved by the Health Sciences Ethics Committee of the Federal University of Rio de Janeiro (CEUA 188/13). All animals received humane care in compliance with the principles of laboratory animal care formulated by the National Society of Medical Research and the Guide for the Care and Use of Laboratory Animals prepared by the National Academy of Sciences, USA.

A total of 50 female C57BL/6 mice (8–12 weeks old) were divided into two groups: silica (SIL), in which animals received an intratracheal injection of 20 mg of silica in 50 μL of saline, and control (CTRL), in which the animals received 50 μL of saline intratracheally. After 15 days, the SIL group was further randomly divided into four groups: Sal (intratracheal injection of 50 μL of PBS), AD-MSC (dose of 100,000 AD-MSCs in 50 μL of PBS), EV5 (EVs obtained from 100,000 AD-MSCs for 24 h, also in 50 μL of PBS), and EV6 (10× higher EV dose than EV5).

### Cell culture

Male C57BL/6 mice (weight 20–25 g, 8 weeks old) were used as donors. MSCs from adipose tissue (epididymal fat pad) were obtained as previously described [25]. A brief description of the isolation procedure and culture conditions is given in Additional file 1.

At the third passage, approximately ten million cells were characterized as MSCs through flow cytometry and induction of differentiation into osteoblasts and chondroblasts as previously described [26]. Cells from the third to fifth passage were used for EV isolation and instillation. For direct instillation, cells were detached with trypsin, washed, and re-suspended in PBS. For in vitro internalization essays, lung fibroblasts were obtained from healthy C57BL/6 mice and cultivated in DMEM containing 1% antibiotic solution, 10% fetal bovine serum (FBS) and 15 mM HEPES for up to 2 weeks [27]. MH-S cells were purchased from ATCC (#CRL2019) and cultivated in RPMI 1640 with 10% FBS, 1% penicillin-streptomycin (10,000 U/mL, Thermo Fisher Scientific, USA) and 5 mM 2-mercaptoethanol according to the vendor’s instructions. RAW264.7 cells were exposed to silica for 2 h (100 μg/mL) followed by treatment with EVs or regular medium for 24 and 48 h.

### EV isolation

AD-MSCs were kept in exosome-free medium (with the same proportion of FBS, previously ultracentrifuged without dilution at 100,000×*g* overnight to remove bovine EVs [28]) at 70–90% confluence. After 24 h, the conditioned medium was removed, and EVs were enriched as follows: centrifugation at 300×*g* for 10 min, supernatant centrifugation at 3000×*g* for 20 min, and final supernatant ultracentrifugation at 100,000×*g* for 2 h (modification from Théry et al. [29]).The pellet was washed and ultracentrifuged with PBS once and then re-suspended in PBS for downstream applications. The methods used for characterization of EVs are given in Additional file 1.

### In vitro and in vivo distribution of EVs

AD-MSCs were stained with Vybrant DiI (Life Technologies, USA) before isolation of EVs. For in vitro internalization experiments, MH-S cells and primary fibroblasts were incubated with 3 × 10^8^ stained vesicles per well in 12-well cell culture plates for 24 h, washed with PBS, fixed in methanol, and mounted with ProLong Gold Antifade Mountant with DAPI DNA dye (Life Technologies, USA). The slides were imaged in a Zeiss LSM 710 confocal microscope at 200× and 400× magnification. For flow cytometry analysis, cells were washed and re-suspended in PBS after incubation and used without fixation.

For in vivo distribution experiments, stained EVs were delivered intratracheally in 50 μL of PBS in 5 C57BL/6 female mice, 15 days after instillation of 20 mg of silica. The lungs were collected 5 min after the instillation, snap frozen in liquid nitrogen, embedded in optimal cutting temperature medium (Tissue-Tek OCT compound, Electron Microscopy Sciences, Fisher Scientific, USA), and cryosectioned into 10 μm slices. Slides were mounted with ProLong Gold/DAPI as above and imaged at 200× magnification with a Zeiss fluorescence microscope.

### Histology

A laparotomy was done immediately after the determination of lung mechanics (as described in Additional file 1) and heparin (1000 IU) was injected intravenously into the vena cava. The trachea was clamped at end expiration, and the abdominal aorta and vena cava were sectioned, yielding a massive hemorrhage that quickly killed the animals. The left lung was then removed, fixed in 3% buffered formaldehyde and embedded in paraffin. Slices were cut (4-μm thick) and stained with hematoxylin–eosin. Lung histology analysis was performed with an integrating eyepiece with a coherent system consisting of a grid with 100 points and 50 lines (known length) coupled to a conventional light microscope (Olympus BX51, Olympus Latin America-Inc., Brazil). The area fraction of granuloma was measured with ImageJ v1.48 software (NIH, USA). The number of mononuclear and polymorphonuclear cells in pulmonary tissue and inside the granuloma was determined using the point-counting technique across 10 random, non-coincident microscopic fields [30]. For the assessment of collagen fiber deposition, slices were stained using the Picrosirius method [31]. The fraction of area occupied by collagen fibers (stained in red) in relation to total tissue area was quantified using Image Pro Plus version 5.1 (Media Cybernetics, USA).

### ELISA

For protein isolation, the right lobes of the lungs were snap frozen in liquid nitrogen and kept at − 80 °C until analysis. Protein was extracted from tissue homogenates using RIPA buffer. Levels of interleukin (IL)1-β, transforming growth factor (TGF)-β, tumor necrosis factor (TNF)-α, and IL-6 in lung tissue were measured by ELISA using matched antibody pairs from PrepoTech (Rocky Hill, NJ, USA) and R&D Systems (Minneapolis, MN, USA), according to the manufacturers’ instructions. Protein levels of TNF-α and TGF-β were also measured in cell supernatants using the same methods.

### Immunohistochemistry

The total number of macrophages was quantified separately in alveolar septa and granuloma through reaction with monoclonal antibody F4/80 rat anti-mouse (AbD Serotec). The antibody was detected using a secondary antibody labeled with peroxidase (Histofine mouse MAX PO, Nichirei Biosciences, Japan) followed by the chromogen substrate, diaminobenzidine (liquid DAB, Dakocytomation, USA). Thirty microscopic fields were randomly selected, avoiding vessels and bronchi, using a digital camera (Evolution, Media Cybernetics, USA) coupled to a light microscope (Eclipse 400, Nikon, Japan), and a computer with graphical interface software (Q-Capture 2.95.0, Silicon Graphics, USA). High-quality images were captured and the number of labeled cells was divided by the total number of cells per field.

### Statistical analysis

For comparison among experimental groups of animals and among different storage conditions of EVs, one-way ANOVA followed by Tukey’s multiple comparison test was applied. When comparing two different sets of EVs, the unpaired t test was used. To screen for changes in the size distribution of vesicles, in addition to modes and mean sizes, mean interquartile ranges were also compared using an unpaired t test. All analyses were performed with GraphPad Prism 6 for Windows (GraphPad Software, La Jolla California, USA). Statistical significance was set at *p* < 0.05.

## Results

### EV isolation and characterization

**Fig. 1:**
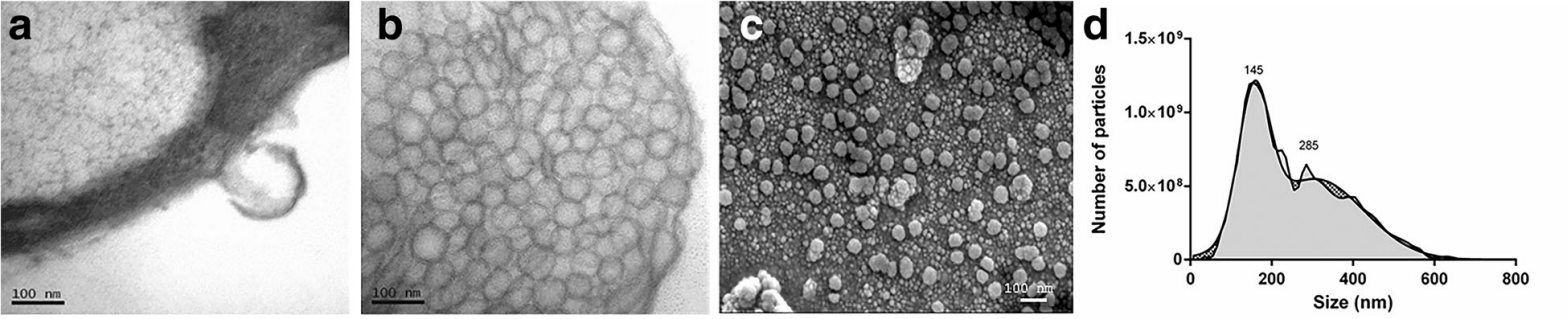
Ultrastructural analysis of the AD-MSC and EV fraction and nanoparticle tracking analysis of the EV fraction. **a, b** Transmission electron microscopy demonstrates the presence of bulges on the cell membrane, indicating possible microvesicles in AD-MSCs. **c** Scanning electron microscopy technique of sequential centrifugation-enriched EV fractions of AD-MSC supernatant shows spherical structures ranging from 50 to 100 nm in diameter and the presence of agglomerates. **d** Size distribution (diameter) of EVs obtained by nanoparticle tracking analysis. Representation of the average from six different samples, with six different videos taken per sample. Curves indicate two Gaussian fittings, and numbers indicate diameter modes

We observed release of EVs from AD-MSCs using transmission electron microscopy (Fig. 1a, b). After sequential centrifugation and ultracentrifugation of the conditioned media [29], the final fraction obtained (which should be enriched in smaller EVs) was observed by scanning electron microscopy (Fig. 1c). Spherical structures under 150 nm in diameter were prevalent in the sample. Larger amorphous structures that suggest aggregation were observed. When measured by nanoparticle tracking analysis (Fig. 1d), the vesicle size distribution was bimodal with mode diameters of 145 nm and 285 nm. This size distribution also indicates the presence of larger populations of vesicles, which is expected for a sample containing vesicular and non-vesicular moieties. The mean size was around 250 nm, in agreement with what was described for human AD-MSC EVs by Lopatina and colleagues [32] using a similar NTA technique. We did not detect EVs larger than 1000 nm, indicating the absence of relevant amounts of apoptotic bodies in our samples. EVs were positive for common EV surface markers: CD63, CD81, Lamp1, and CD9 (Additional file 2); 10^6^ cells (EV6 dose) produced on average 3.84 × 10^9^ EVs, with a standard deviation of 1.92 × 10^9^. No differences in size distribution or concentration were observed when comparing EVs from cells at different passages (data not shown). The fraction of vesicles obtained did not present 18S or 28S ribosomal RNA fractions, indicating negligible cytosolic contamination, but small RNAs were detected. Total RNA concentration was around 50 ng/10^9^ vesicles. Protein content was around 40 μg/10^9^ vesicles.

To explore whether different cell culture conditions could increase the yield of EVs, we stressed the cells by serum deprivation. FBS deprivation has advantages from a translational standpoint for the elimination of a xenogeneic component from production protocols. On the other hand, serum deprivation can stress cells and change their physiological state, consequently affecting EV composition and production, which could then affect the therapeutic outcome (positively or negatively) [33]. When cells were deprived of FBS for 24 h, the yield of EVs was significantly decreased (Additional file 3).

### EV uptake by lung cell lineages and in vivo distribution in the lungs

**Fig. 2:**
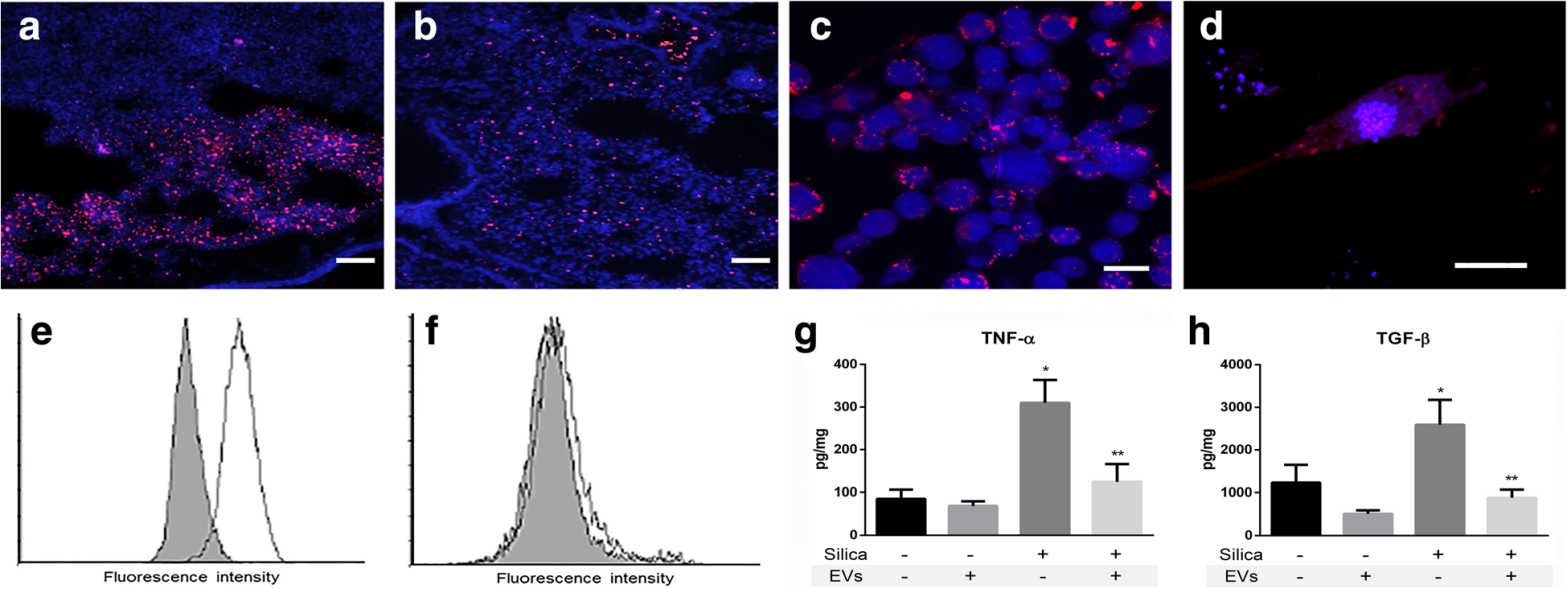
Microscopy images showing (**a**) EV distribution (red) in the lung parenchyma immediately after local instillation; (**b**) EV distribution (red) in the lung parenchyma 30 min after local instillation; EV uptake (red) by (**c**) MH-S (alveolar macrophages) × 200 and (**d**) primary lung fibroblast (× 400, scale bars represent 20 μm). **e** Flow cytometry representative results of EV uptake (Vybrant DiI staining) by MH-S (alveolar macrophages) after incubation for 24 h. **f** Flow cytometry representative results of EV uptake (Vybrant DiI staining) by lung primary fibroblasts after incubation for 24 h. Gray areas indicate negative controls. TNF-α (**g**) and TGF-β (**h**) concentration (per total amount of protein) in the conditioned medium of cultured macrophages stimulated with silica with or without addition of EVs; bars indicate means ± SEM, *n* = 4, *Significantly different from unstimulated group. **Significantly different from stimulated group without EVs, *p* < 0.05

To test whether our target lung cells were able to internalize EVs in culture, we stained EVs with a lipophilic dye and observed the uptake of vesicles in vitro in primary lung fibroblasts and a lineage of alveolar macrophages. Macrophages displayed efficient uptake of vesicles (Fig. 2c, e), as expected considering the optimal size of the vesicles for phagocytosis by these cells. Uptake was neither halted nor increased by previous activation of macrophages with silica particles (data not shown). When exposed to silica in the presence of EVs, macrophages of the RAW lineage produced lower amounts of TNF-α and TGF-β (Fig. 2g, h), suggesting inhibition of activation. Uptake of vesicles by fibroblasts was observed in foci for the same concentration of vesicles in vitro*,* with a lower percentage of cells containing the dye (Fig. 2d, f).

In addition, we investigated the distribution of vesicles in vivo after intratracheal instillation of EVs (Fig. 2a, b). The vesicles reached the alveoli and were especially concentrated closer to the airways. Both healthier areas and areas with intense inflammation presented fluorescence. We followed this uptake from 5 min to 1 h, observing the same fluorescence intensity throughout the parenchyma.

### AD-MSCs and EVs modulate silica-induced lung inflammation

**Fig. 3:**
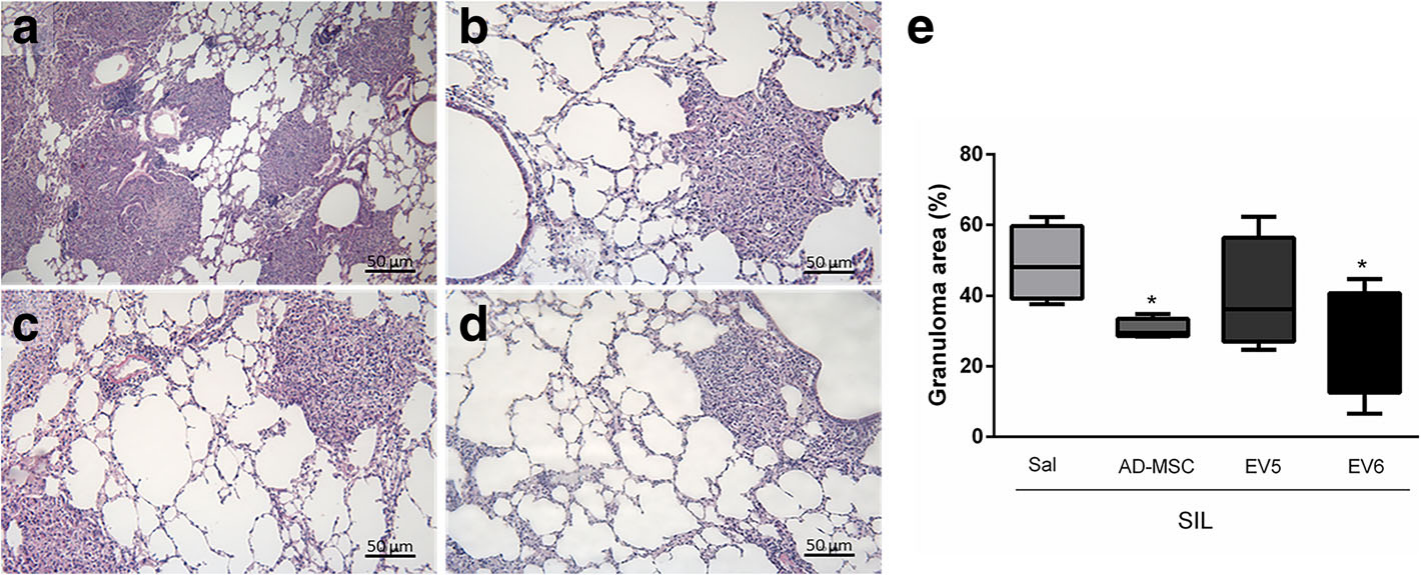
Representative photomicrographs of the lung parenchyma of animals from the silica group (**a**) and the SIL group treated with cells (**b**) or EVs in two different doses: EV5 (**c**) and EV6 (**d**). Slices were stained with hematoxylin–eosin staining. Arrows indicate granulomas, 200×. **e** Fraction of area of granuloma in the lungs, quantified by analysis of photomicrographs of 5 animals per group, represented as a percentage of the total area of lung tissue. *Significantly different from SIL-Sal, *p* < 0.05

**Fig. 4:**
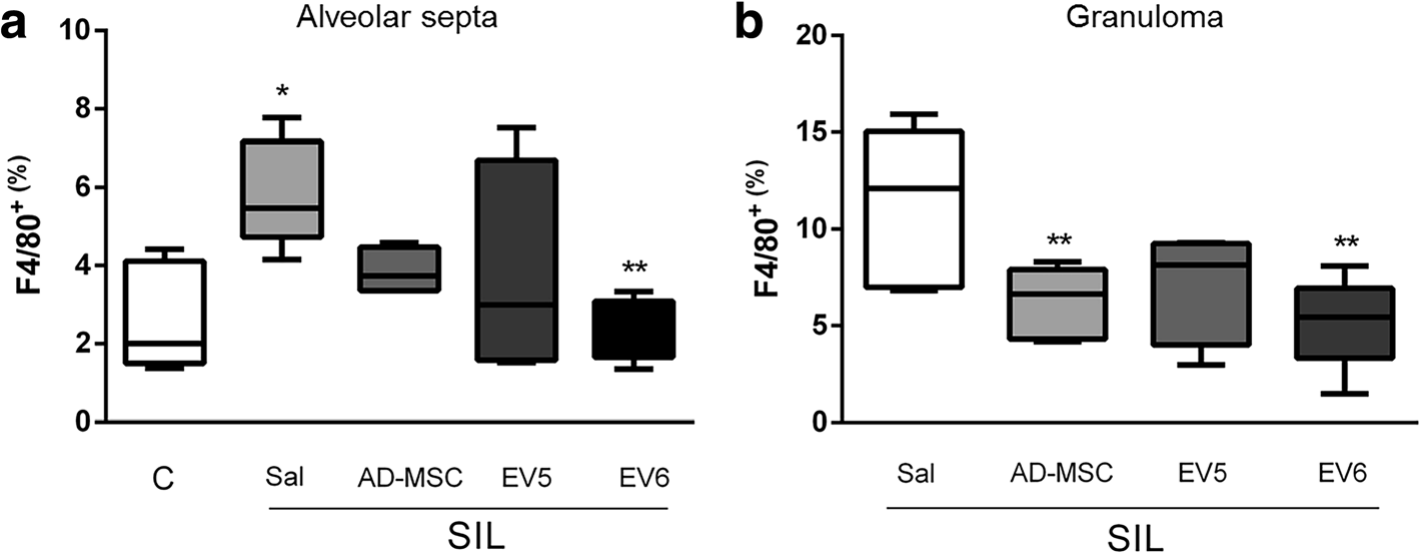
Percentage of F4/80 positive cells (macrophages) in alveolar septa (**a**) and inside the granulomatous tissue (**b**). Box plots are representations (min to max) of 20 random field measurements from 5 animals per group. *Significantly different from control group (labeled C). **Significantly different from SIL-Sal group (*p* < 0.05)

**Fig. 5:**
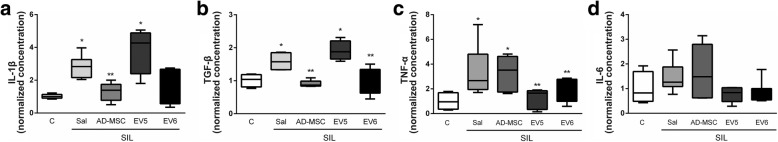
Relative measurement of (**a**) IL-1β, (**b**) TGF-β, (**c**) TNFα, (**d**) IL-6 in the lung tissue. Values were normalized by control levels. *Significantly different from control group (labeled C). **Significantly different from SIL-Sal group (*p* < 0.05)

A single dose of AD-MSCs or EVs in the dose equivalent to the production of one million cells for 24 h (EV6) significantly reduced the fraction area of granuloma in the lungs of SIL animals (Fig. 3). Total tissue cellularity was significantly reduced inside the granuloma with the EV treatments (Table 1) but not in the alveolar septa (data not shown). Moreover, the number of macrophages was reduced by the treatment with AD-MSCs or EV6, both in the alveolar septa and inside the granuloma (Fig. 4a and b), thus showing modulation of the inflammatory response by both the cells and EVs. IL1-β and TGF-β expression in the lung tissue was decreased in the same groups (Fig. 5a, b) but not in the group that received the lower dose of EVs, whereas TNF-α was reduced with both concentrations of EVs but not by the cells (Fig. 5c). IL-6 was not significantly affected by silica or by the treatments (Fig. 5d).

**Table 1:** Tissue cellularity within silicotic nodules of animals from the silica groups

	MN (%)	PMN (%)	Total (%)
Sal	39.97 ± 4.337	1.747 ± 2.074	43.10 ± 6.574
AD-MSC	34.94 ± 1.505	1.193 ± 1.039	36.13 ± 1.453
EV5	33.47 ± 2.506^a^	0.7568 ± 0.2735	34.22 ± 2.375^a^
EV6	32.01 ± 4.106^a^	1.209 ± 0.8275	34.18 ± 5.553^a^

Values are means ± standard deviation of 5 animals

*MN* mononuclear cells, *PMN* polymorphonuclear cells

^a^Significantly different from Sal group

### Effects of AD-MSCs and EVs in lung remodeling and function

**Fig. 6:**
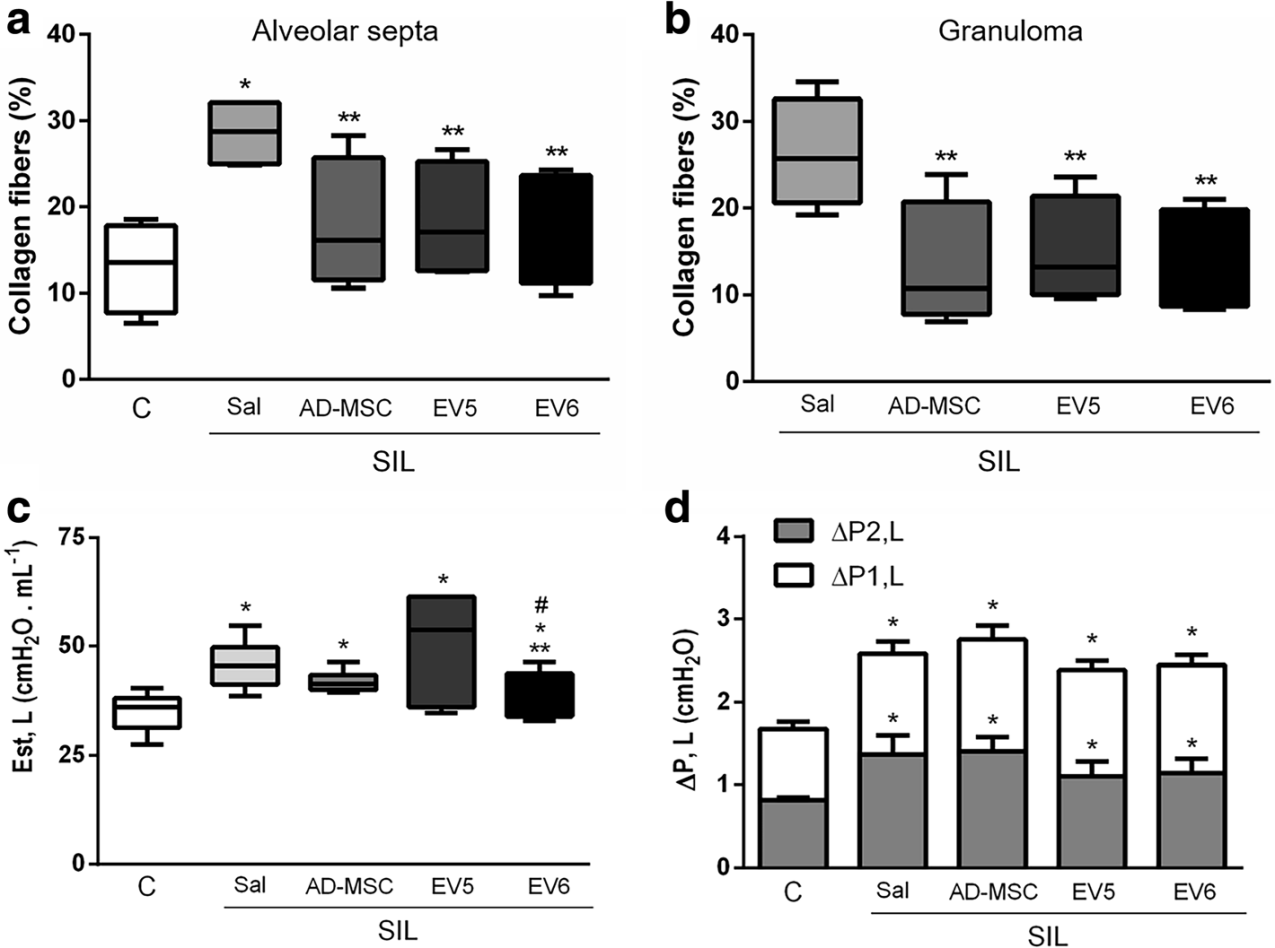
Impact of silica and treatments with MSCs or EVs in lung remodeling and lung mechanics. **a, b** Box plot representation of collagen fiber content (min to max) in the alveolar septa (**a**) or inside the granuloma (**b**). Values indicate measurements from 10 random fields of 5 animals per group *Significantly different from control group (labeled C). **Significantly different from SIL-Sal group (*p* < 0.05). **c** Static lung elastance values in box plot representation (min to max). **d** Stacked bars represent viscoelastic (ΔP2) and resistive (ΔP1) pressures. Fifteen measurements were taken from each animal, and 6–10 animals were analyzed per group. *Significantly different from control group (labeled C). **Significantly different from SIL-Sal group (*p* < 0.05)

Lung fibrosis is the most important consequence of silica inhalation. SIL groups presented increased collagen deposition compared with CTRL groups. Both interstitial fibrosis and the progressive accumulation of fibers around granulomatous tissue were reduced by the treatments with either AD-MSCs or EVs (Fig. 6a, b). Despite the decrease in collagen fibers with both AD-MSCs and EVs, only a mild effect on lung static elastance was observed. Treatment with the higher dose of EV6 was able to reduce this parameter significantly but without complete reversion to control values (Fig. 6c). No significant differences were observed in viscoelastic or resistive pressure coefficients with any of the treatments compared with untreated silicotic animals (Fig. 6d).

### EV stability under different conditions

**Fig. 7:**
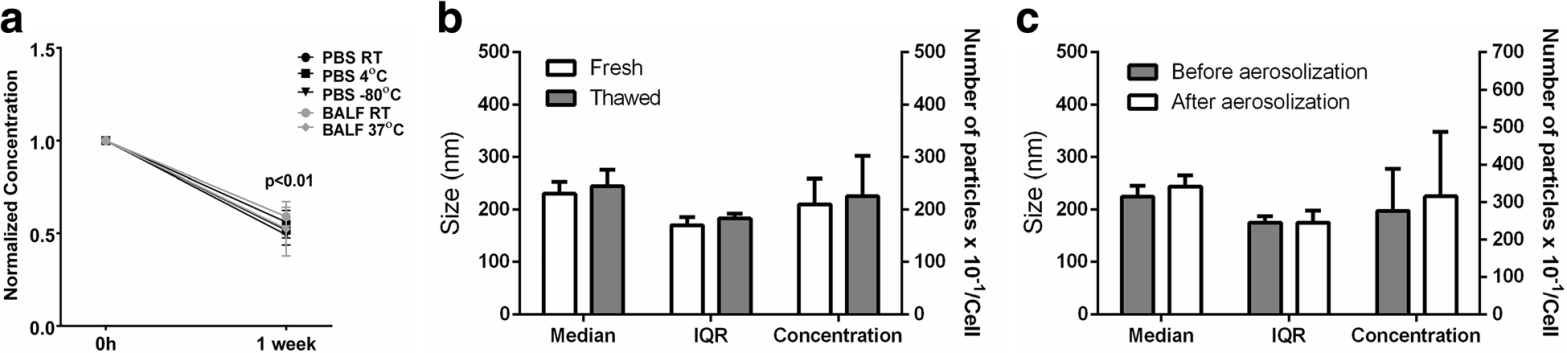
Mean interquartile range, median, and concentration of vesicles (bars/symbols represent means ± SD of 4–6 independent experiments). **a** Concentration of vesicles per cell normalized by the initial values of freshly obtained vesicles. **b** Freshly obtained conditioned medium versus thawed medium after 3 weeks of storage at − 80 °C were used to isolate EVs. **c** Each sample of EVs was measured before and after aerosolization with a microsprayer syringe

When considering a translational approach to the use of EVs, knowledge of EV stability under different storage conditions is needed. We measured the concentration and size distribution of AD-MSC EVs after 1 week of storage in different conditions: (1) room temperature in saline solution; (2) in bronchoalveolar lavage fluid (BALF) collected in PBS; (3) saline solution at 4 °C; (4) BALF at 37 °C; (5) saline solution at − 80 °C. No changes in the mean interquartile range or in the median diameter were observed after 1 week under the different conditions. All storage conditions caused a decrease in concentration of about 50%, but without significant changes in size (Fig. 7a). However, when the conditioned medium was frozen up to 3 weeks before ultracentrifugation, no changes in size distribution or concentration were observed (Fig. 7b). We then tested the ability of EVs to endure aerosolization, a property essential for the proposed local delivery of EVs to treat respiratory illnesses. We used a microsprayer syringe system to generate an aerosol from suspended EVs. There were no changes in the size parameters or concentration of EVs (Fig. 7c). This suggests that no major disruption or aggregation of vesicles occurs during aerosolization.

## Discussion

Because the use of bone-marrow-derived mesenchymal cell therapy has yielded positive results in a variety of lung diseases, including silicosis and lung fibrosis induced by silica, as we previously reviewed [34], we aimed to test whether AD-MSCs are able to reproduce these effects, and if AD-MSC EVs are sufficient, providing a possible alternative to the use of cells. AD-MSCs have been shown to exert immunomodulation in inflammatory diseases [35–37] and have several advantages over other MSCs: (1) higher yield of cells per gram of tissue; (2) higher proliferation rate then bone-marrow-derived MSCs; and (3) easier access to the cell source, because adipose tissue is often discarded after elective surgical procedures [38–40].

In the present study, treatment with AD-MSCs or EVs was performed after the onset of the chronic features of silicosis, triggered by a single intratracheal dose of silica 15 days before the treatment. At this point, there is extensive fibrosis and development of mature silicotic nodules [41, 42]. This study design has the advantage of providing better insight into a therapeutic approach, in contrast with other studies that show effects of EVs in prophylactic treatments [13, 43]. To establish the dose of EVs to be used for treatment, we took previous cell therapy studies as reference. In pre-clinical studies, the doses often range from 10^5^ to 10^6^ cells. We then tested two doses of EVs arrived at by back-calculating to these numbers [44–47].

Two weeks after treatment, we observed a decrease in inflammatory cell counts in the tissue of all treated animals. The extent of granulomatous tissue was significantly decreased by both AD-MSCs and the higher dose of EVs, but not with the lower dose of EVs. There was also a significant reduction in the number of macrophages inside the granulomas of these two groups; however, only the treatment with the higher dose of EVs was able to reduce the number of macrophages in the alveolar septa. The decrease in macrophage count is in agreement with other studies on cell therapy with bone-marrow-derived cells [8, 10], and, as in these studies, was accompanied by a decrease in IL-1β and TGF- β in the lung tissue. These factors play prominent roles in the development of lung fibrosis, triggering lung fibroblast activation, fibrocyte recruitment, and epithelial-to-mesenchymal transition [48].

Treatment with both AD-MSCs and EVs, regardless of the dose of the latter, was able to halt or reverse collagen fiber deposition, albeit without complete return to control values. Interestingly, the treatment with EVs at the lower dose was able to reduce fibrosis despite not having the same efficiency in halting the inflammation parameters compared with the other groups. That could be an indication of a more pronounced earlier immunomodulatory effect that has been borne out by endpoint analyses. This finding indicates that these treatments efficiently promote amelioration of fibrosis, and that the mechanisms for this effect may not be exclusively dependent on sustained immunosuppression.

Furthermore, silica administration led to a significant increase in lung static elastance, in agreement with previous studies from our group [9, 10]. Treatment with the higher dose of EVs was able to ameliorate this parameter, but none of the treatments were able to completely restore the elastance to control values, which may be explained by the persistence of silicotic nodules and fibrosis.

Our results show that both AD-MSCs and their EVs are able to promote therapeutic effects in this late-stage model of silicosis. Administration of EVs at the higher concentration yielded outcomes comparable with the cells themselves in this therapy, promoting enhanced impact on lung mechanics and macrophage infiltration. Nevertheless, additional studies are required: (1) to better understand the mechanisms underlying the therapeutic effects of EVs, (2) to assess the effects of higher doses or multiples instillations, (3) to evaluate whether these effects may be even greater when cells are previously stimulated.

Therapeutic administration of EVs has been explored as a novel approach in several models of disease for their inherent therapeutic value or for their efficacy as vectors for gene therapy [18, 49, 50]. Nevertheless, important questions remain regarding the general properties of EVs, such as stability and uptake kinetics, and their use in the disease-specific therapeutic setting, including dose selection (equivalent to cell therapy doses in the number of cells used over a specific time, per kilogram, single or multiple doses, etc.), administration route (local or systemic), and timing of delivery (continuously, before or after the onset of the disease, or after aggravation of specific symptoms, etc.).

In this work, we isolated a heterogeneous fraction of EVs from the culture supernatant of primary murine AD-MSCs. This fraction contained EVs of different sizes, and thus possibly originated from different intracellular compartments. These vesicles presented favorable characteristics for clinical translation, such as a simple and reproducible isolation protocol and size and concentration stability (after storage in different storage conditions and after aerosolization). When stored for 1 week after ultracentrifugation, there was a significant decrease in the concentration of the EV samples, regardless of storage temperature. An explanation for this, besides possible sample degradation, is adherence of the EVs to the surface of storage tubes [51]. A decrease in EV concentration was not observed when they were stored in conditioned media before ultracentrifugation.

We cannot exclude the possibility that only a subset of the EVs introduced into the animals were responsible for the observed effects. Substantial scale-up of EV production beyond what we could achieve in this study would be needed to examine the potential contributions of EV subtypes or co-purifying substances for silicosis treatment. Future studies could combine larger-scale production with additional purification steps (density gradients or chromatography) to assess the performance of different fractions of EVs or to reduce bodies such as large protein complexes that may co-purify with EVs [51, 52].

Our group has shown that intratracheal instillation of bone marrow cells, compared with systemic routes, has greater therapeutic effects in murine models of asthma and emphysema [26, 53]. In addition, intratracheal instillation, when compared with a broader distribution throughout the organism by systemic exposure routes, offers two important advantages: (1) immediate access to the target tissue; and (2) ability to adjust and optimize the EV dose in the target tissue. Assessing the biodistribution and bioavailability of EVs after instillation requires challenging techniques and may depend upon vesicle size and cellular type of origin [54, 55]. In this study, we demonstrate that isolated EVs stained with a lipophilic dye successfully reach the lung parenchyma. This approach has the limitation of lack of specificity of the dye, making it impossible to confirm if the vesicles are intact or to estimate their half-life in the tissue (the dye could have been transferred to other lipid membranes) but bypasses the possible selective staining of one specific kind of vesicles among the heterogeneous sample.

Using the same staining strategy, we show that alveolar macrophages present high uptake of EVs compared with lung primary fibroblasts. This might be due to the expression of CD44 receptor on macrophages, because this receptor has been implicated in the cellular uptake of MSC-derived EVs in previous studies [14, 50]. The incorporation of EVs by alveolar macrophages, even after the phagocytosis of silica particles, is important for the immunosuppressive and cytoprotective effects of these vesicles, and macrophages that received EVs produced less TNF-α and TGF-β upon stimulation with silica. Macrophages are central to the pathogenesis of silicosis, secreting pro-fibrotic cytokines and recruiting inflammatory cells [56]. It has been demonstrated that BM-MSC-derived EVs can modulate gene expression and metabolism of macrophages through the delivery of miRNAs and mitochondria, decreasing the activation of the transcription factor NFκB and the TLR signaling pathway, and decreasing inflammation in the lung of mice 3 days after instillation of a lower dose of silica [43]. Taken together, the evidence suggests that these cells might be important targets in MSC-derived EV treatments.

## Conclusion

In conclusion, this work suggests that AD-MSC-based therapies, both directly with the cells and more importantly with the use of EVs derived from these cells, should be further investigated and adapted for the clinical setting as an alternative treatment of silicosis.

## Additional files

Additional file 1: Supplementary Methods. (DOCX 16 kb)

Additional file 2: **Figure S1.** Representative flow cytometry analysis. Fluorescence intensity for the marked beads are represented by the empty curves, and gray areas represent fluorescence intensity of beads incubated with the negative controls (isotypes). One hundred thousand events were analyzed per experiment. (TIF 1676 kb)

Additional file 3: **Figure S2.** Mean interquartile range, median and yield of vesicles per cell obtained from medium depleted of FBS (Without FBS) or containing EV-free FBS (with FBS). *Significantly different from “Without FBS”, *p* < 0.05. (TIF 99 kb)
