# Supplementary material for: Therapeutic effects of adipose-tissue-derived mesenchymal stromal cells and their extracellular vesicles in experimental silicosis
Source: Respir Res. 2018 May 29;19:104. doi: 10.1186/s12931-018-0802-3 (PMC5975461; doi:10.1186/s12931-018-0802-3)
Supplement: Supplementary file 1 — Supplementary Methods. (DOCX 16 kb) [file 12931_2018_802_MOESM1_ESM.docx]

**Additional file 1**

METHODS

***AD-MSC isolation and culture***

Tissues were collected, rinsed in PBS, transferred to a Petri dish, and cut into small pieces. The dissected pieces (around 0.2-0.8 cm^3^) were washed with PBS, and subsequently digested with collagenase type I (1 mg/mL in PBS) for 30-40 minutes at 37°C. After digestion, fresh medium was added and the suspension was centrifuged at 400 g for 10 minutes at room temperature (RT). The pellets were re-suspended in DMEM containing 1% antibiotic solution (Invitrogen, CA, USA), 20% FBS and 15 mM HEPES, seeded in T25 flasks (4 mL per flask), and incubated at 37°C in a humidified atmosphere containing 5% CO2. On day 3 of culture, the medium was changed and non-adherent cells were removed. Adherent cells reaching 80% confluence were passaged with 0.25% trypsin-EDTA solution (Gibco, NM, USA).

***EV size and stability assessments***

EV size distribution was measured by nanoparticle tracking analysis (NTA) with a NS500 device (NanoSight, Malvern) equipped with a SCMOS camera and the software version NTA 3.0. Six videos of 30 seconds each were analyzed for each sample, using the same camera level and a detect threshold set at 3. For stability assessments, the EVs were kept on PBS or Broncho alveolar lavage fluid obtained from C57BL/6 mice and filtered through a 50KDa concentration filter (Amicon, Millipore). The number of detected particles in the undiluted sample after filtration fell below detection threshold of NTA. To measure stability after aerosolization, each sample was equally divided in two parts: One measured directly through NTA and another one that was sprayed with a microsprayer syringe (IA-1C S/M–551 Model, Penn-Century, Inc, Philadelphia, PA, USA) prior to measurement. Concentration measurements were corrected by cell counts.

***Electron microscopy***

Cells were fixed in 2.5% glutaraldehyde in 0.1M sodium cacodylate buffer (pH=7.4) for 40 min and post-fixed with a solution of 1% OsO_4_, 0.8% potassium ferricyanide and 2.5mM CaCl_2_ in the same buffer for 20 min at room temperature. dehydrated in an ascending acetone series and embedded in PolyBed 812 resin. Ultrathin sections were stained with uranyl acetate and lead citrate; these sections were examined under a Jeol JEM1011 transmission electron microscope (Tokyo, Japan). Alternatively, for scanning electron microscopy, dehydrated samples were dried by the critical point method with CO_2_, mounted on aluminum stubs, and coated with a 20-nm-thick gold layer, and examined under a Jeol JSM6390LV or Jeol JSM-7001F scanning electron microscope. All ultrastructural analysis were performed at Plataforma de Microscopia Eletrônica in IOC, Fiocruz.

***Flow cytometry***

Flow cytometry of EVs was performed as described in Thery at al., 2006. Briefly, EV protein content was measured using BCA. EVs samples were incubated with latex beads for 2h, followed by incubation with glycine 1M for 15min. The beads were washed and incubated with antibodies against CD9 (ebiosciences), CD63(BD Pharmingen), CD81 (BD Pharmingen) and Lamp1 (ebiosciences). Isotypes were used as negative controls. Samples were analyzed with the instruments BD Accuri or BD FACSCalibur.

***Lung mechanics***

Thirty days after the intratracheal instillation of silica, animals were sedated (diazepam 1 mg intraperitoneally (ip), anesthetized (thiopental sodium 20 mg/kg ip), tracheotomized, paralyzed (vecuronium bromide, 0.005 mg/kg intravenously), and ventilated with a constant flow ventilator (Samay VR15; Universidad de la República, Montevideo, Uruguay) with the following parameters: frequency of 100 breaths/min, tidal volume (VT) of 0.2 ml, and fraction of inspired oxygen of 0.21. The anterior chest wall was surgically removed and a positive end-expiratory pressure of 2 cmH_2_O applied. Airflow and tracheal pressure (Ptr) were measured (Burburan et al., 2007). Lung mechanics were analyzed by the end-inflation occlusion method (Bates et al., 1988). In an open chest preparation, Ptr reflects transpulmonary pressure (PL). Briefly, after end-inspiratory occlusion, there is an initial fast drop in PL (ΔP1) from the preocclusion value down to an inflection point (Pi), followed by low pressure decay (ΔP2), until a plateau is reached. This plateau corresponds to the elastic recoil pressure of the lung (Pel). ΔP1 selectively reflects the pressure used to overcome the airway resistance. ΔP2 reproduces the pressure spent by stress relaxation, or viscoelastic properties of the lung, together with a small contribution of *pendelluft*. Static lung elastance (Est) was determined by dividing Pel by VT. Lung mechanics measurements were performed 10 times per animal. All data were analyzed using ANADAT data analysis software (RHT- InfoData, Inc., Montreal, Quebec, Canada).
