# Supplementary figures and images for: Therapeutic effects of adipose-tissue-derived mesenchymal stromal cells and their extracellular vesicles in experimental silicosis
Source: Respir Res. 2018 May 29;19:104. doi: 10.1186/s12931-018-0802-3 (PMC5975461; doi:10.1186/s12931-018-0802-3)

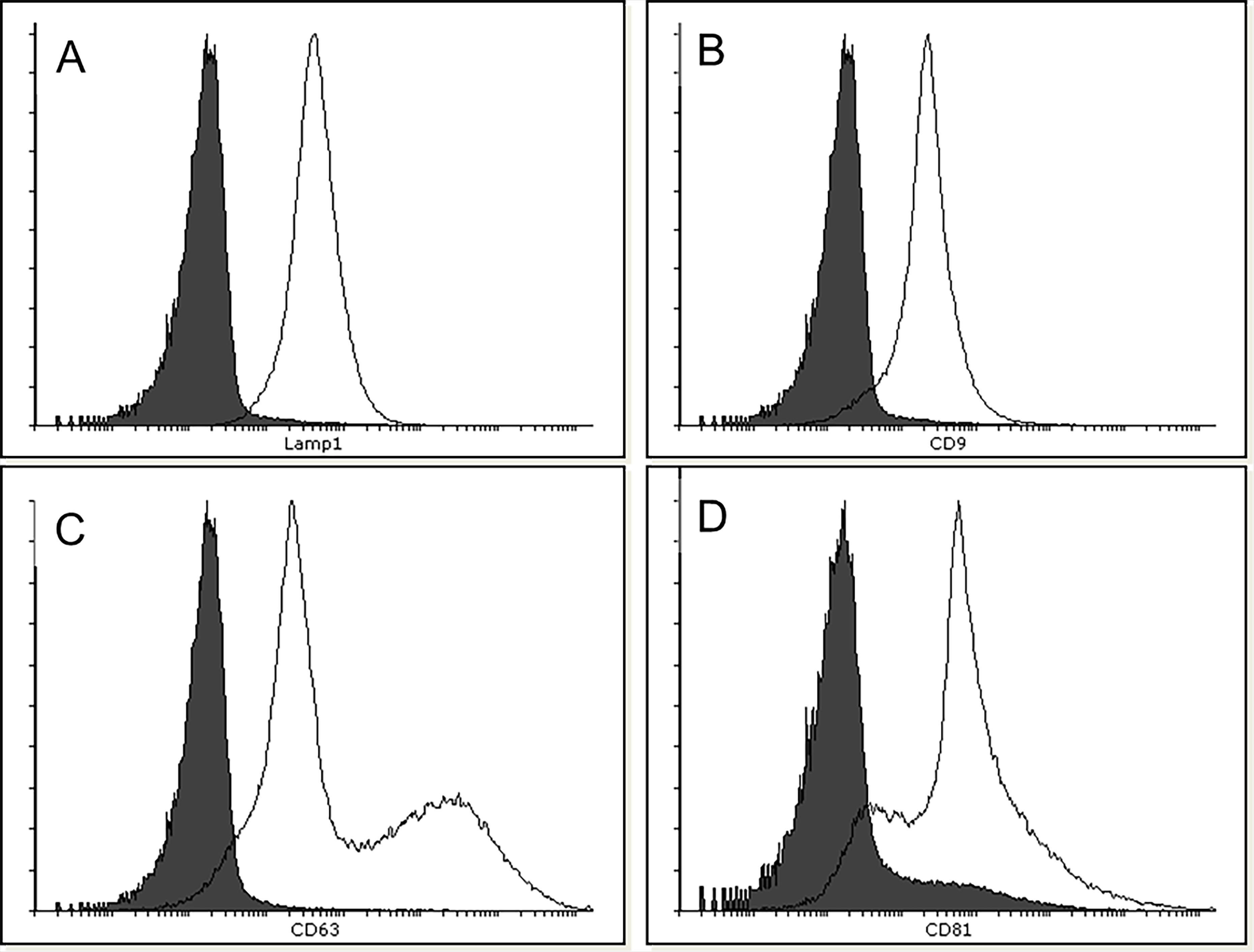

Supplement: Supplementary file 2 — Figure S1. Representative flow cytometry analysis. Fluorescence intensity for the marked beads are represented by the empty curves, and gray areas represent fluorescence intensity of beads incubated with the negative controls (isotypes). One hundred thousand events were analyzed per experiment. (TIF 1676 kb) [file 12931_2018_802_MOESM2_ESM.tif]
